# Efficacy of Immune Checkpoint Inhibitors in Combination With Platinum‐Based Doublet Chemotherapy for Extensive‐Stage Small‐Cell Lung Cancer Patients With Eastern Cooperative Oncology Group‐Performance Status 2–3: A Single‐Institution Retrospective Study

**DOI:** 10.1002/cam4.71136

**Published:** 2025-09-10

**Authors:** Kosuke Sakai, Shigeru Ishii, Shin Yokosuka, Tomoyuki Takahashi, Yuichiro Kawano, Hiroaki Nishimura, Yoshiki Kuwabara, Maiko Sasaki‐Toda, Yumiko Ogawa‐Kobayashi, Satoshi Kikuchi, Yusuke Hirata, Hiroyuki Kyoyama, Gaku Moriyama, Nobuyuki Koyama, Kazutsugu Uematsu

**Affiliations:** ^1^ Department of Pulmonary Medicine Saitama Medical Center, Saitama Medical University Saitama Japan

**Keywords:** anti‐PD‐L1 antibodies, atezolizumab, durvalumab, extensive‐stage small‐cell lung cancer, immune checkpoint inhibitor, performance status

## Abstract

**Background:**

The prognosis of small‐cell lung cancer (SCLC) remains poor, particularly in patients with extensive‐stage SCLC. The IMpower133 and CASPIAN trials revealed the efficacy of immune checkpoint inhibitors (ICIs) in extensive‐stage SCLC patients with good performance status (PS). The aim of this study was to investigate the efficacy of ICIs in patients with poor PS.

**Patients and Methods:**

Patients with extensive‐stage SCLC who visited Saitama Medical Center, Saitama Medical University (Kawagoe, Japan) (September 2019–December 2022) were enrolled and followed up until February 2024. Objective response rate (ORR) and overall survival (OS) were compared between patients who received platinum‐based doublet chemotherapy with an ICI (atezolizumab or durvalumab; ICI group) and those treated with platinum‐based doublet chemotherapy alone (non‐ICI group). Results were stratified by the Eastern Cooperative Oncology Group performance status (ECOG‐PS) (i.e., 0–1 and 2–3).

**Results:**

A total of 74 patients were included in the study (median OS: 327 days). In patients with ECOG‐PS 0–1, ORR was 76.5% and 56.5% in the ICI group (*n* = 17) and non‐ICI group (*n* = 23), respectively; OS was 406 and 379 days, respectively. In patients with ECOG‐PS 2–3, ORR was 93.3% and 56.3% in the ICI group (*n* = 15) and non‐ICI group (*n* = 16), respectively; OS was 446 days and 169 days, respectively. This evidence indicates that the addition of an ICI significantly improved OS (*p* = 0.00661) and enhanced ORR.

**Conclusion:**

In patients with extensive‐stage SCLC and ECOG‐PS 2–3, the addition of atezolizumab or durvalumab to platinum‐doublet chemotherapies improved the ORR, resulting in a better prognosis. These findings suggest that chemoimmunotherapy may be a feasible treatment option beyond the ideal clinical trial populations, addressing an unmet clinical need.

**Abbreviations**
AUC: area under the curve
CI: confidence interval
CR: complete response
ECOG‐PS: Eastern Cooperative Oncology Group‐performance status
ES: extensive‐stage
ICI: immune checkpoint inhibitor
NA: not applicable
ORR: objective response rate
OS: overall survival
PD: progressive disease
PD‐L1: programmed cell death‐ligand 1
PR: partial response
SCLC: small‐cell lung cancer
SD: stable disease

## 1 Introduction

Small‐cell lung cancer (SCLC) accounts for approximately 10%–15% of all lung cancer cases [1]. At the time of initial diagnosis, 60%–70% of patients with SCLC present with distant metastasis due to the rapid dissemination of cancer cells to other organs and lymph nodes [2]. In the 1970s, the prognosis for SCLC was poor, with 1‐ and 5‐year survival rates of 23% and 3.6%, respectively [3]. Since the 1980s, the standard chemotherapy regimen for extensive‐stage SCLC (ES‐SCLC) has included cisplatin and etoposide, which had been shown to be equivalent to cyclophosphamide, epirubicin, and vincristine [4]. For elderly patients or those with poor general conditions, cisplatin was replaced by carboplatin based on the Japan Clinical Oncology Group 9702 clinical trial. The median overall survival (OS) was 10.6 months in patients treated with split‐dose cisplatin and etoposide compared to 9.9 months in those treated with carboplatin and etoposide [5]. In the early 2000s, treatments prolonged the median OS from 11.3 months to 15.2 months [6]. In the 2010s, the 1‐ and 5‐year survival rates were 30.8% and 6.8%, respectively [3]. Cardiovascular or liver comorbidities, a poor Eastern Cooperative Oncology Group‐performance status (ECOG‐PS) of 3 or 4, and extensive‐stage disease leading to noncurative treatment were identified as independent factors adversely affecting the prognosis of patients with SCLC [7].

In July 2014, the Pharmaceuticals and Medical Devices Agency (Japan) approved nivolumab for the treatment of malignant melanoma. Thereafter, cancer immunotherapies have been widely utilized and included in the treatment of lung cancer. Atezolizumab and durvalumab (anti‐programmed cell death‐ligand 1 [PD‐L1] antibodies termed immune checkpoint inhibitors [ICIs]) were approved in August 2019 and August 2020, respectively, for the treatment of SCLC. The IMpower133 clinical trial revealed that the addition of atezolizumab to treatment with carboplatin and etoposide extended the median OS compared to carboplatin, etoposide, and placebo (12.3 vs. 10.3 months, respectively) [8]. One year later, the CASPIAN clinical trial demonstrated the efficacy of adding durvalumab to the combination of cisplatin or carboplatin and etoposide in the treatment of ES‐SCLC. The median OS was 13.0 and 10.3 months in the durvalumab and control groups, respectively; this prolonged median OS was associated with improvement in the objective response rate (ORR) from 58% to 68% [9]. However, in both trials, only patients in good general condition (ECOG‐PS 0–1) were enrolled; therefore, there is insufficient evidence regarding the effect of ICI addition to conventional chemotherapy for patients with ES‐SCLC in poor general condition.

Patients with SCLC often present with poor general condition early due to rapid tumor progression. However, chemotherapies for SCLC may improve symptoms or organ function owing to their efficacy for rapid tumor shrinkage. In patients with SCLC in poor general condition, it is expected that chemotherapy agents will result in clinical improvement. Nevertheless, this is not the case for patients with non‐small‐cell lung cancer in a similar condition [10, 11]. To expand effective treatment options, it is crucial to evaluate the efficacy and tolerability of ICIs in SCLC patients with ECOG‐PS 2–3. Several real‐world studies, ranging from small to large scale, have investigated the clinical outcomes of chemoimmunotherapy in patients with ES‐SCLC, including those with poor general condition. However, most of these studies focused on comparing patients who did or did not meet the eligibility criteria of pivotal trials such as IMpower133 or CASPIAN, and many have reported that patients with good performance status tend to have better outcomes than those with poor status, although the findings are not entirely consistent across studies [12, 13, 14]. In contrast, it remains unclear whether the addition of ICIs provides a meaningful benefit specifically for ES‐SCLC patients with poor general condition, particularly when compared to those who received chemotherapy alone. At our facility, atezolizumab or durvalumab has been used in combination therapies to improve outcomes, even in patients with ECOG‐PS 2–3. Based on our clinical experience, we hypothesized that the combination of cancer immunotherapy, including atezolizumab or durvalumab, with conventional chemotherapy would improve the prognosis of patients with SCLC in poor general condition. Therefore, we performed a retrospective study to investigate whether the combination of ICIs with chemotherapy may improve treatment outcomes in SCLC patients with poor conditions.

## 2 Patients and Methods

### 2.1 Patients

The enrolled patients in this study: (1) visited Saitama Medical Center, Saitama Medical University from September 1, 2019, to December 31, 2022; (2) underwent pretreatment examination including computed tomography, positron emission tomography‐computed tomography, brain magnetic resonance imaging, bone scintigraphy, and abdominal ultrasonography; (3) had pathologically confirmed ES‐SCLC without other types of cancer; and (4) had not undergone thoracic radiotherapy.

### 2.2 Procedures

Clinical information was retrospectively collected from the electronic medical records at Saitama Medical Center. The ECOG‐PS was obtained based on the description of the patient's functional status in the initial history and physical examination. Response to treatment was classified as complete response (CR), partial response (PR), stable disease (SD), and progressive disease (PD), according to the Response Evaluation Criteria in Solid Tumors, version 1.1. ORR was calculated as the proportion of patients with a CR or PR. OS was defined as the time from treatment initiation to death due to any cause. Follow‐up was conducted until February 29, 2024. This study included patients who were lost to follow‐up to maintain continuity in our hospital. OS and ORR to treatment were estimated and stratified by ECOG‐PS, based on whether or not patients received an ICI (atezolizumab or durvalumab).

### 2.3 Statistical Analysis

The distribution of characteristics (sex, ECOG‐PS, clinical stage according to the Union for International Cancer Control 8th edition, and ORR to the treatment) between patients who received treatment with an ICI and those who did not was evaluated using Fisher's exact test. Distribution of age was evaluated by the Mann–Whitney *U* test. Survival time was estimated by the Kaplan–Meier method, and differences in OS were evaluated by the log‐rank test. The factors associated with OS were estimated using Cox proportional hazards regression analysis. In this analysis, patients aged ≥ 71 and ≤ 70 years were classified as elderly and young, respectively. Patients with ECOG‐PS 0–1 and 2–3 were categorized as having good and poor PS, respectively. The factors associated with ORR or decision by the attending physicians to use ICI concomitantly were estimated using logistic regression analysis. Statistical analyses were performed using the EZR software [15], with the level of significance set at *p* value < 0.05.

### 2.4 Ethics Statement

The study was approved by the ethics board of Saitama Medical Center, Saitama Medical University (approval number: 2023–056).

## 3 Results

### 3.1 Patients and Treatments

**FIGURE 1:**
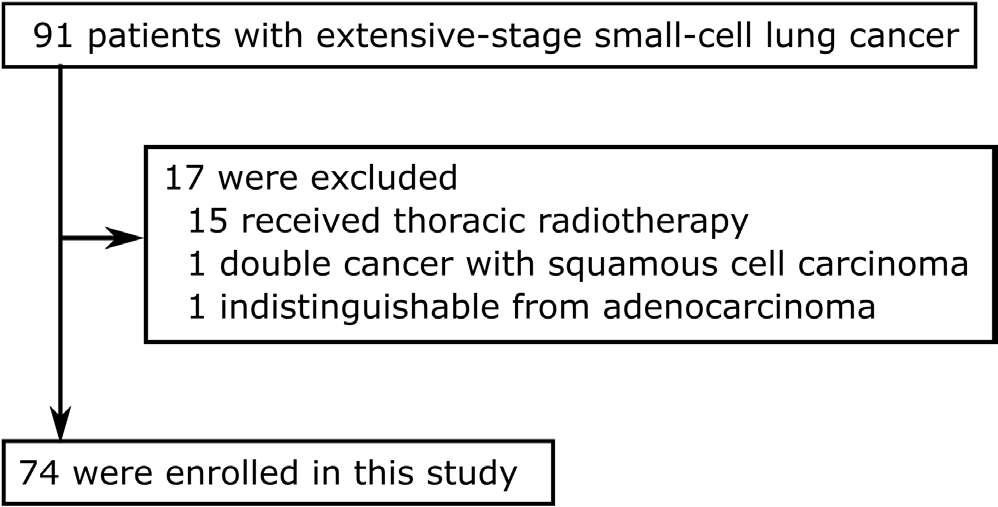
CONSORT diagram for the selection of patients with extensive‐stage small‐cell cancer.

During the study period, 91 patients were treated for ES‐SCLC at Saitama Medical Center, Saitama Medical University. Of those, 17 patients were excluded from this analysis because they did not meet the enrollment criteria: 15 patients underwent thoracic radiotherapy; one patient suffered from double cancer with squamous cell carcinoma; and one patient had an indistinguishable diagnosis from adenocarcinoma. Consequently, 74 patients were included in this single‐institution retrospective study (Figure 1).

**FIGURE 2:**
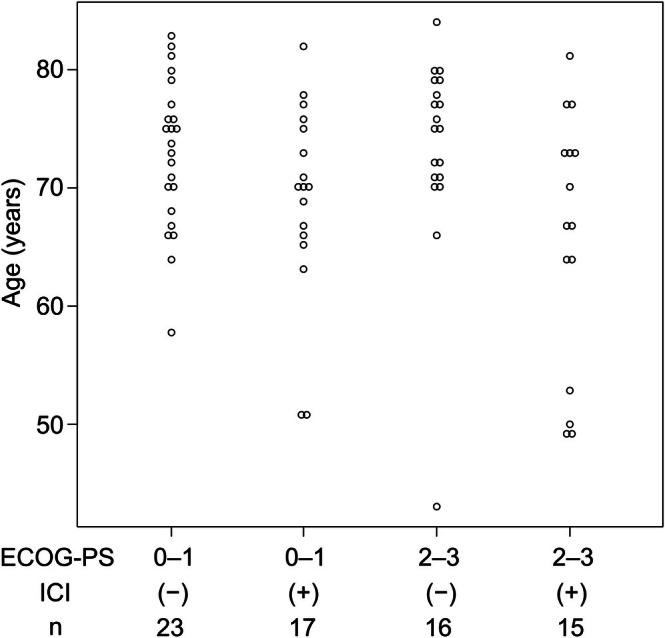
Dot chart of age, stratified by the Eastern Cooperative Oncology Group‐performance status (ECOG‐PS), and the use (+) or nonuse (−) of an immune checkpoint inhibitor (ICI).

The patients were divided into those who received an ICI (durvalumab or atezolizumab) in combination with platinum and etoposide as first‐line therapy (ICI group: *n* = 32) and those who did not receive ICIs as first‐line therapy (non‐ICI group: *n* = 42) (Table 1). There were no significant differences in sex, ECOG‐PS, and clinical stage between the groups; however, patients in the ICI group were significantly younger than those in the non‐ICI group (*p* = 0.0065). This tendency remained even in patients with an ECOG‐PS of 0 or 1, as well as those with an ECOG‐PS of 2 or 3 (Figure 2). The median observation period was 218 days for all patients, 274 days for those with ECOG PS 0–1, and 177 days for those with ECOG PS 2–3.

**TABLE 1:** Patient characteristics.

	All (*n* = 74)	ICI (−) (*n* = 42)	ICI (+) (*n* = 32)	*p*
Sex				0.765
Male	60 (81.1%)	35 (83.3%)	25 (78.1%)	
Female	14 (18.9%)	7 (16.7%)	7 (21.9%)	
Age (years)				0.0065
Median	72	75	70	
Range	43–84	43–84	49–82	
Performance status				0.154
0	3 (4.1%)	2 (4.8%)	1 (3.1%)	
1	37 (50.0%)	21 (50.0%)	16 (50.0%)	
2	15 (20.3%)	5 (11.9%)	10 (31.3%)	
3	16 (21.6%)	11 (26.2%)	5 (15.6%)	
4	3 (4.1%)	3 (7.1%)	0	
Stage (UICC 8th edition)				0.204
IIIA	1 (1.4%)	1 (23.8%)	0	
IIIB	4 (5.4%)	2 (4.8%)	2 (6.3%)	
IIIC	3 (4.1%)	3 (7.1%)	0	
IVA	12 (16.2%)	9 (21.4%)	3 (9.4%)	
IVB	54 (73.0%)	27 (64.3%)	27 (84.4%)	

Abbreviations: ICI, immune checkpoint inhibitor; UICC, Union for International Cancer Control.

In the non‐ICI group (*n* = 42), 39 patients received carboplatin and etoposide, while three received cisplatin and etoposide. In the ICI group (*n* = 32): 22 patients received carboplatin, etoposide, and atezolizumab, 15 of whom (68.2%) continued to receive atezolizumab as maintenance therapy; nine received carboplatin, etoposide, and durvalumab, four of whom (44.4%) continued to receive durvalumab; and one received cisplatin, etoposide, and durvalumab, followed by durvalumab therapy (Table 2). The number of cycles of ICI administration as maintenance treatment was as follows: 1 (*n* = 5); 2 (*n* = 8); 3 (*n* = 5); 6 (*n* = 1); and 59 (*n* = 1). In the ICI group, chemotherapies administered after the first‐line chemotherapy were as follows: second‐line chemotherapy (*n* = 22) including re‐challenge with carboplatin and etoposide (*n* = 6; of whom, five and one patients received atezolizumab and durvalumab, respectively, in the first line), cisplatin and irinotecan (*n* = 2), and amrubicin (*n* = 14); third‐line chemotherapy (*n* = 15) including cisplatin and irinotecan (*n* = 2), irinotecan (*n* = 2), amrubicin (*n* = 7), and nimustine and paclitaxel (*n* = 4); and fourth‐line chemotherapy (*n* = 6) including cisplatin and irinotecan (*n* = 1), irinotecan (*n* = 1), nogitecan (*n* = 1), and nimustine and paclitaxel (*n* = 3). In the non‐ICI group, those chemotherapies were as follows: second‐line chemotherapy (*n* = 16), including re‐challenge with carboplatin and etoposide (*n* = 6), amrubicin (*n* = 5), and nimustine and paclitaxel (*n* = 5); third‐line chemotherapy (*n* = 10) including amrubicin (*n* = 2), nogitecan (*n* = 2), and nimustine and paclitaxel (*n* = 6); and fourth‐line chemotherapy with nogitecan (*n* = 2).

**TABLE 2:** First‐line chemotherapy.

	First‐line chemotherapy	*n*	ICI maintenance *n* (%)
ICI (−)	Carboplatin, etoposide	39	
	Cisplatin, etoposide	3	
ICI (+)	Carboplatin, etoposide, atezolizumab	22	15 (68.2%)
	Carboplatin, etoposide, durvalumab	9	4 (44.4%)
	Cisplatin, etoposide, durvalumab	1	1 (100.0%)

Abbreviation: ICI, immune checkpoint inhibitor.

### 3.2 Efficacy

The ORR to first‐line chemotherapy was 67.6% in all patients; in the non‐ICI and ICI groups, the ORR was 54.8% and 84.4%, respectively. In patients with ECOG‐PS 0–1, the ORR was 65.0% (56.5% and 76.5% in the non‐ICI and ICI groups, respectively). In patients with ECOG‐PS 2–3, the ORR was 74.2% (56.3% and 93.3% in the non‐ICI and ICI groups, respectively). In patients with ECOG‐PS 2–3, the ORR was significantly improved. This significant difference led to a statistically significant improvement in ORR in the ICI group across all patients, even though the ICI group in patients with ECOG‐PS 0–1 did not show significant improvement in ORR compared to the non‐ICI group (Table 3). Among multiple variables, including age, ECOG‐PS, and the addition of ICIs, logistic regression analysis identified the addition of ICIs as the sole factor associated with better ORR (Table 4).

**TABLE 3:** Objective response rate to first‐line chemotherapy.

	Complete response	Partial response	Stable disease	Progressive disease	Overall response rate	*p*
All (*n* = 74)	7 (9.5%)	43 (58.1%)	15 (20.3%)	9 (12.2%)	67.6%	
ICI (−) (*n* = 42)	1 (2.4%)	22 (52.4%)	12 (28.6%)	7 (16.7%)	54.8%	0.0144
ICI (+) (*n* = 32)	6 (18.8%)	21 (65.6%)	3 (9.4%)	2 (6.3%)	84.4%	
PS 0–1 (*n* = 40)	3 (7.5%)	23 (57.5%)	10 (25.0%)	4 (10.0%)	65.0%	
ICI (−) (*n* = 23)	1 (4.3%)	12 (52.2%)	8 (34.8%)	2 (8.7%)	56.5%	0.397
ICI (+) (*n* = 17)	2 (11.8%)	11 (64.7%)	2 (11.8%)	2 (11.8%)	76.5%	
PS 2–3 (*n* = 31)	4 (12.9%)	19 (61.3%)	4 (12.9%)	4 (12.9%)	74.2%	
ICI (−) (*n* = 16)	0	9 (56.3%)	3 (18.8%)	4 (25.0%)	56.3%	0.0226
ICI (+) (*n* = 15)	4 (26.7%)	10 (66.7%)	1 (6.7%)	0	93.3%	

Abbreviation: ICI, immune checkpoint inhibitor.

**TABLE 4:** Multivariate analysis.

	*p*	Variance inflation factor
Factors associated with overall response rate		
Age	0.3920	1.1543
ECOG‐PS	0.6860	1.0090
Receiving ICI	< 0.01	1.1448
Factors associated with overall survival		
Age	0.1123	
ECOG‐PS	< 0.01	
Receiving ICI	0.3321	
Response (CR or PR) to the first‐line chemotherapy	< 0.01	
Factors associated with decision to use ICI		
Age	< 0.01	1.0342
ECOG‐PS	0.7530	1.0271
Duration from ICI approval	< 0.05	1.0591

Abbreviations: CR, complete response; ECOG‐PS, Eastern Cooperative Oncology Group‐performance status; ICI, immune checkpoint inhibitor; PR, partial response.

**FIGURE 3:**
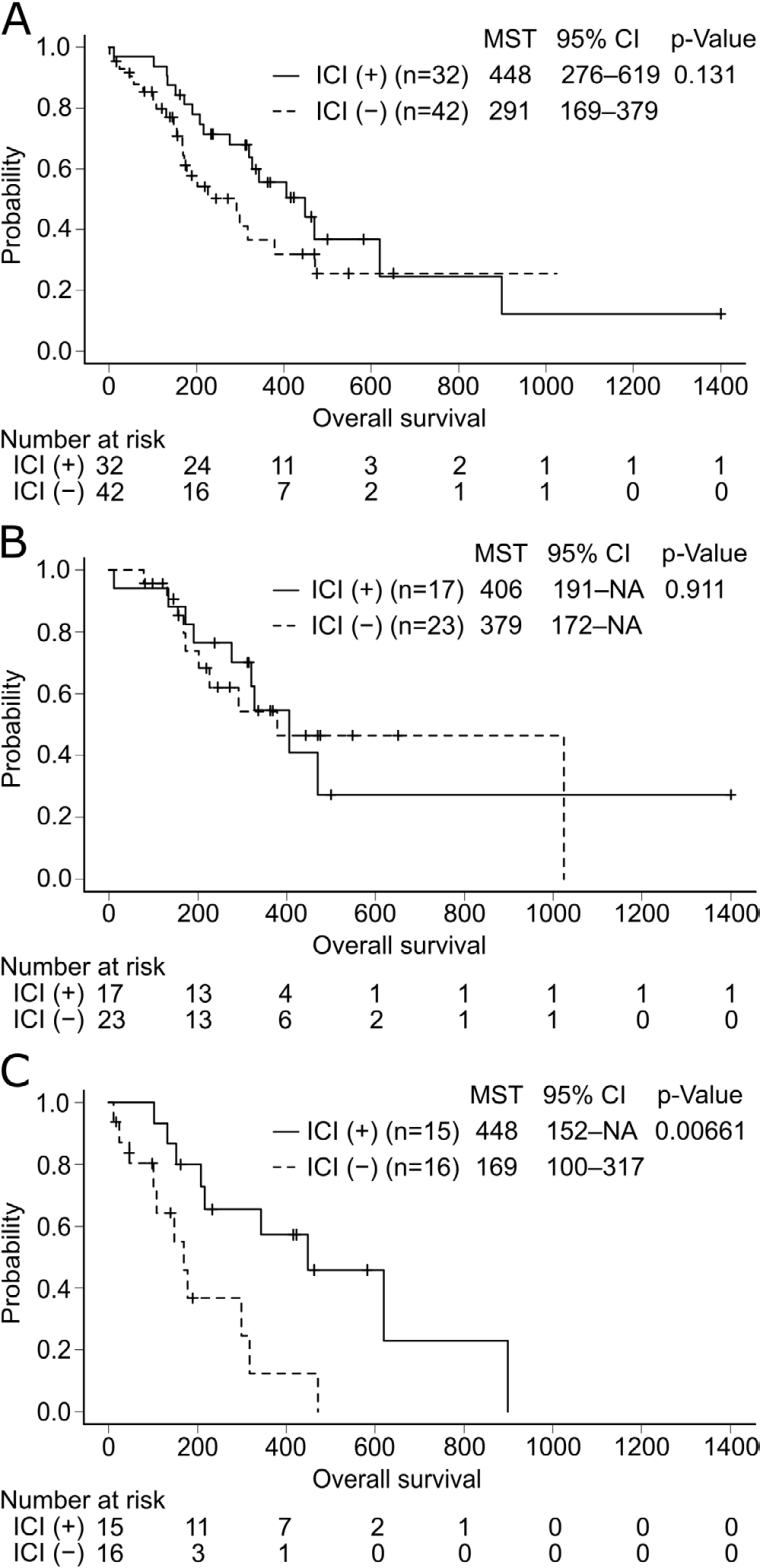
Kaplan–Meier curves of overall survival (OS). (A) OS in all enrolled patients with extensive‐stage small‐cell lung cancer (ES‐SCLC). Patients were divided into those who received chemotherapy (cisplatin or carboplatin, and etoposide) with an immune checkpoint inhibitor (ICI), that is, ICI (+) group, and those who received chemotherapy without an ICI, that is, ICI (−) group. (B, C) OS in patients with ES‐SCLC with Eastern Cooperative Oncology Group performance status (ECOG‐PS) of 0–1 (B) and 2–3 (C). CI, confidence interval; MST, median survival time; NA, not applicable.

The median OS for all patients was 327 days (95% confidence interval [CI]: 216–470 days). In the ICI and non‐ICI groups, median OS was 448 days (95% CI: 276–619 days) and 291 days (95% CI: 169–379 days), respectively (*p* = 0.131) (Figure 3A). In patients with ECOG‐PS 0–1, the median OS was 406 days (95% CI: 191 days–not applicable [NA]) and 379 days (95% CI: 172 days–NA), respectively (*p* = 0.911) (Figure 3B). In patients with ECOG‐PS 2–3, the median OS was 448 days (95% CI: 152 days–NA) and 169 days (95% CI: 100–317 days), respectively (*p* = 0.00661). The Cox proportional hazards regression analysis revealed that a good PS and a CR or PR to the first‐line chemotherapy contributed to the prolongation of OS (Table 4). The addition of ICIs did not affect the OS in patients with good PS and all patients. Nonetheless, it significantly prolonged the OS in patients with poorer PS (Figure 3C).

**FIGURE 4:**
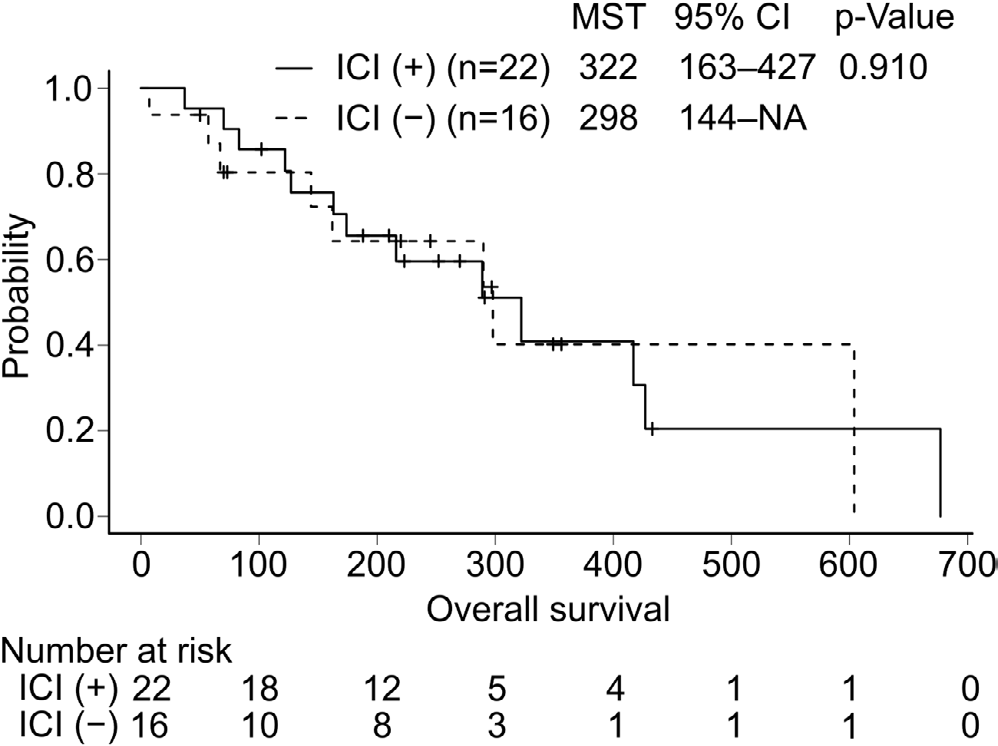
Kaplan–Meier curves of overall survival time from the induction of the second‐line chemotherapy in patients with extensive‐stage small‐cell lung cancer. Patients were divided into two groups based on whether or not they received an immune checkpoint inhibitor (ICI) as first‐line therapy: ICI (+) group and ICI (−) group. CI, confidence interval; MST, median survival time; NA, not applicable.

In 38 patients who received second‐line treatment, the ORR to the second‐line treatment was 43% and 54% in the non‐ICI group and ICI group, respectively (*p* = 0.134) (Table 5); the OS following initiation of the second‐line chemotherapy was 322 days and 298 days, respectively (*p* = 0.910) (Figure 4). The ORR to second‐line treatment and OS after the initiation of second‐line treatment did not differ between the ICI combination group and the non‐ICI group in the initial treatment.

**TABLE 5:** Objective response rate to second‐line chemotherapy.

	Complete response	Partial response	Stable disease	Progressive disease	*p*
All (*n* = 38)	4 (10.5%)	15 (39.5%)	12 (31.6%)	7 (18.4%)	
ICI (−) (*n* = 16)	2 (12.5%)	5 (31.3%)	8 (50.0%)	1 (6.3%)	0.134
ICI (+) (*n* = 22)	2 (9.1%)	10 (45.5%)	4 (18.2%)	6 (27.3%)	

Abbreviation: ICI, immune checkpoint inhibitor.

### 3.3 Adverse Events and Discontinuation

In the non‐ICI group and ICI group, the prevalence of severe (grade ≥ 3) neutropenia, anemia, thrombocytopenia, and febrile neutropenia was 85.7% versus (vs.) 62.5%, 23.8% vs. 3.1%, 9.5% vs. none, and 9.5% vs. none, respectively. The prevalence of severe adverse events according to ECOG‐PS is shown in Table 6. In nine patients, ICI administration was discontinued due to immune‐related adverse events (Table 7).

**TABLE 6:** Adverse events (grade ≥ 3).

	ICI (−) (*N* = 42)				ICI (+) (*N* = 32)			
ECOG‐PS	0–1		≥ 2		0–1		≥ 2	
	(*N* = 23)		(*N* = 19)		(*N* = 17)		(*N* = 15)	
Adverse events	Grade 3	Grade 4	Grade 3	Grade 4	Grade 3	Grade 4	Grade 3	Grade 4
Neutropenia	7 (30.4%)	14 (60.9%)	4 (21.1%)	11 (57.9%)	5 (29.4%)	6 (35.3%)	6 (40.0%)	3 (20.0%)
Anemia	1 (4.3%)	2 (8.7%)	6 (31.6%)	1 (5.3%)	1 (5.9%)	0	0	0
Thrombocytopenia	1 (4.3.%)	1 (4.3%)	0	2 (10.5%)	0	0	0	0
Febrile neutropenia	2 (8.7%)	0	2 (10.5%)	0	0	0	0	0
Diarrhea	0	0	0	0	0	0	1 (6.7%)	0
Hypopituitarism	0	0	0	0	1 (5.9%)	0	0	0
Pneumonitis	0	0	0	0	1 (5.9%)	0	0	0
Rash	0	0	0	0	1 (5.9%)	0	1 (6.7%)	0

Abbreviations: ECOG‐PS, Eastern Cooperative Oncology Group performance status; ICI, immune checkpoint inhibitor.

**TABLE 7:** Immune‐related adverse events leading to discontinuation of ICI administration.

ECOG‐PS	ICI	Number of cycles of ICI administration	Carboplatin dosage	Etoposide dosage (mg/m^2^)	Adverse events	Grade	Response	Overall survival (days)
1	Atezolizumab	6	AUC 5	100	Rash	3	PR	238
1	Durvalumab	3	AUC 5	80	Arthritis	2	SD	369
1	Atezolizumab	5	AUC 5	100	Pneumonitis	3	PR	315
1	Atezolizumab	6	AUC 5	100	Hypopituitarism	3	CR	499
2	Atezolizumab	7	AUC 5	80	Diarrhea	2	PR	233
2	Atezolizumab	4	AUC 5	100	Diarrhea	3	CR	898
2	Atezolizumab	5	AUC 5	100	Encephalitis	2	PR	162
2	Atezolizumab	1	AUC 5	100	Rash	3	PR	448
3	Durvalumab	2	AUC 4	60	Hypothyroidism	2	CR	152

Abbreviations: AUC, area under the curve; CR, complete response; ECOG‐PS, Eastern Cooperative Oncology Group‐performance status; ICI, immune checkpoint inhibitor; PR, partial response; SD, stable disease.

A 77‐year‐old woman with a chest wall metastasis and ECOG‐PS 3 received chemotherapy with a reduced dose of carboplatin (area under the curve [AUC]: 4) and etoposide (60 mg/m^2^) due to her poor condition; durvalumab was added from the second cycle. Administration of durvalumab was discontinued after the fourth cycle due to grade 2 hypothyroidism. Chemotherapy dose reduction due to poor PS was not required in other patients.

## 4 Discussion

Pivotal studies on the use of chemotherapies combined with ICIs (e.g., anti‐PD‐L1 antibodies) for ES‐SCLC included only patients with ECOG‐PS 0–1 [8, 9]. However, in practice, ICIs have been administered to patients with ECOG‐PS 2–3 in combination with platinum‐based doublet chemotherapy. The present retrospective study showed that the addition of ICIs to first‐line chemotherapy significantly prolonged the OS and improved the ORR in patients with a poorer general condition (i.e., ECOG‐PS 2–3) compared to patients treated with the ICI‐excluded chemotherapy. Furthermore, the combination regimen did not improve the ORR and OS in patients with ECOG‐PS 0–1. The patients with ECOG‐PS 2–3 who were treated with ICIs tended to be relatively younger. Nevertheless, the Cox proportional hazards regression analysis revealed that OS prolongation was associated with CR or PR, but not with age. Additionally, logistic regression analysis revealed that the combination regimen improved the ORR. These results suggest that the addition of ICIs improved the ORR in patients with ECOG‐PS 2–3, resulting in prolongation of survival.

The results of a prospective, dose‐adjusted study on the safety profile of durvalumab plus carboplatin and etoposide in patients with ES‐SCLC with ECOG‐PS 2–3 were presented at the 2024 American Society of Clinical Oncology Annual Meeting [16]. This study demonstrated the tolerability with adjustment of dosage and reported that the median OS for patients with ECOG‐PS 2 was 9.5 months with carboplatin of AUC 4 and etoposide of 80 mg/m^2^. In patients with ECOG‐PS 3, the median OS was 5.1 months with carboplatin of AUC 3 and etoposide of 60 mg/m^2^. Except for one patient in whom durvalumab was added later, we did not reduce the dose of carboplatin or cisplatin and etoposide, despite poor ECOG‐PS. In the present study, chemotherapy in combination with ICIs was feasible without an initial dose reduction and achieved a longer median OS of 448 days compared to 169 days for chemotherapy without ICIs.

We acknowledge the following limitations of the present study. Owing to the retrospective and observational nature of this investigation at a single institute, an imbalance in the number of patients with poor prognostic factors may have affected the results. The administration of ICIs was influenced by the familiarity of the attending physician with concomitant ICI use. Based on logistic regression analysis, the duration from the approval of the combination regimen for ES‐SCLC and patient age were identified as factors associated with the decision to use ICIs in combination with chemotherapy (Table 4). Patient age was considered a potential confounding factor in our investigation.

Our investigation suggests that the addition of ICIs to platinum‐doublet chemotherapies prolonged OS in SCLC patients with ECOG‐PS 2–3, although it did not improve OS in those with ECOG‐PS 0–1. The absence of a significant survival benefit in the ECOG PS 0–1 subgroup appears to be inconsistent with the findings of pivotal studies such as the IMpower133 trial and the CASPIAN trial [8, 9]. This discrepancy may be attributable to the following factors. First, the sample size in our study was relatively small, comprising approximately 10% of the populations enrolled in the pivotal studies, which may have limited the statistical power to detect a survival difference. Second, the median overall survival in the non‐ICI group in our study was relatively prolonged (379 days, or 12.6 months), compared with 10.3 months in the control arms of both pivotal trials. In the ICI group, the ORR in patients with ECOG‐PS 0–1 and ECOG‐PS 2–3 was 76.5% and 93.3%, respectively; in the non‐ICI group, these values were 56.5% and 56.3%, respectively. The ORR was better in the ICI group than in the non‐ICI group; however, among patients with ECOG PS 0–1, the improvement in ORR was not statistically significant and did not translate into a better prognosis. Therefore, the combined use of ICI with chemotherapy resulted in tumor shrinkage, which was more beneficial for survival in patients with poor ECOG‐PS than for those with better ECOG‐PS.

## 5 Conclusion

Even in patients with ES‐SCLC with ECOG‐PS 2–3, indicative of poor general condition, administration of atezolizumab or durvalumab in combination with platinum‐doublet chemotherapy was feasible without dosage adjustment. The addition of ICIs may be effective in ES‐SCLC patients with ECOG‐PS 2–3 and improve the response rate in terms of tumor shrinkage, leading to better prognosis. These results highlight a potential treatment opportunity for a patient population often underrepresented in clinical trials.

## Author Contributions

**Kosuke Sakai:** conceptualization, methodology, formal analysis, investigation, resources, data curation, visualization, writing – original draft. **Shigeru Ishii:** resources. **Shin Yokosuka:** resources. **Tomoyuki Takahashi:** resources. **Yuichiro Kawano:** resources. **Hiroaki Nishimura:** resources. **Yoshiki Kuwabara:** resources. **Maiko Sasaki‐Toda:** resources. **Yumiko Ogawa‐Kobayashi:** resources. **Satoshi Kikuchi:** resources. **Yusuke Hirata:** resources. **Hiroyuki Kyoyama:** resources. **Gaku Moriyama:** resources. **Nobuyuki Koyama:** resources, writing – review and editing, supervision. **Kazutsugu Uematsu:** supervision, resources, writing – review and editing.

## Ethics Statement

The study was approved by the ethics board of Saitama Medical Center, Saitama Medical University (approval number: 2023–056).

## Consent

This study is a retrospective study, and patient consent was obtained through the facility's opt‐out procedure.

## Conflicts of Interest

The authors declare the following financial interests/personal relationships which may be considered as potential competing interests. **Kosuke Sakai** received honoraria from AstraZeneca K.K., Chugai Pharmaceutical Co., Nippon Boehringer Ingelheim Co., Novartis Pharma K.K., Pfizer Japan Inc., Eli Lilly Japan K.K., Bristol‐Myers Squibb K.K., Nippon Kayaku Co. Ltd., Merck Biopharma Co. Ltd., and Thermo Fisher Scientific Inc.; and research funding from Eli Lilly Japan K.K. **Tomoyuki Takahashi** received honoraria from AstraZeneca K.K. **Yoshiki Kuwabara** received honoraria from AstraZeneca K.K. and Chugai Pharmaceutical Co. **Yumiko Ogawa‐Kobayashi** received honoraria from Daiichi Sankyo Co. Ltd. **Yusuke Hirata** received honoraria from AstraZeneca K.K. **Hiroyuki Kyoyama** received honoraria from AstraZeneca K.K., Chugai Pharmaceutical Co., Bristol‐Myers Squibb K.K., Nippon Boehringer Ingelheim Co. and Takeda Pharmaceutical Co. Ltd. **Gaku Moriyama** received honoraria from AstraZeneca K.K., Chugai Pharmaceutical Co., Eli Lilly Japan K.K., Merck Biopharma Co. Ltd., and MSD K.K. **Nobuyuki Koyama** received honoraria from AstraZeneca K.K. Chugai Pharmaceutical Co., Nippon Boehringer Ingelheim Co., Novartis Pharma K.K., Eli Lilly Japan K.K., Bristol‐Myers Squibb K.K., Amgen K.K., Nippon Kayaku Co. Ltd., TAIHO Pharmaceutical Co., Daiichi Sankyo Co. Ltd., Takeda Pharmaceutical Co. Ltd. and MSD K.K. **Kazutsugu Uematsu** received honoraria from AstraZeneca K.K., Chugai Pharmaceutical Co., Nippon Boehringer Ingelheim Co., Eli Lilly Japan K.K., Novartis Pharma K.K., TAIHO Pharmaceutical Co., Bristol‐Myers Squibb K.K. and MSD K.K.; and research funding from Chugai Pharmaceutical Co., Nippon Boehringer Ingelheim Co., Novartis Pharma K.K. and TAIHO Pharmaceutical Co. The remaining Authors have no conflicts of interest to declare in relation to this study.

## Data Availability

The data that support the findings of this study are available from the corresponding author upon reasonable request.
